# Synergistic UV and acridine orange mutagenesis enhances polyethylene biodegradation by *Sphingobacterium prati* BS2

**DOI:** 10.3389/fmicb.2026.1730975

**Published:** 2026-03-12

**Authors:** Xiuting Zeng, Piaopiao Li, Wenxuan Zheng, Yihan Zhao, Tingting Zhang, Lei Ma

**Affiliations:** Key Laboratory of Oasis Town and Mountain-Basin System Ecology of XPCC, Key Laboratory of Xinjiang Phytomedicine Resource Utilization of Ministry of Education, College of Life Sciences, Shihezi University, Shihezi, China

**Keywords:** biofilm, bioremediation, polyethylene biodegradation, polymer oxidation, *Sphingobacterium prati*, synergistic mutagenesis

## Abstract

Polyethylene (PE) pollution persists due to its extreme environmental recalcitrance. Microbial degradation offers a promising solution, yet its efficiency in wild-type strains remains limited. To enhance the PE-degrading capability of *Sphingobacterium prati* BS2, this study applied physical (UV irradiation and microwave) and chemical (acridine orange (AO) and hydroxylamine hydrochloride) mutagenesis, followed by directed screening using PE as the sole carbon source. Among the obtained mutants, the UV–AO combined mutant BS2-UA, showing a clear synergistic effect, exhibited the highest positive mutation rate (23.70%) and maintained high stability across successive generations. BS2-UA displayed improved growth performance and biofilm formation, accompanied by pronounced and sustained culture acidification, as well as superior PE degradation, achieving a weight loss of ∼10% over 50 days, which was approximately 50% higher than that of the wild-type strain. Scanning electron microscopy revealed pronounced surface cracks, while Fourier-transform infrared spectroscopy indicated elevated hydroxyl and carbonyl groups, confirming oxidative modification of PE. These findings demonstrate that synergistic mutagenesis is a potent strategy for enhancing the intrinsic plastic-degrading potential of under-explored bacterial genera like *Sphingobacterium*, providing high-performance candidates and insights into systems-level traits underlying effective PE biodegradation.

## 1 Introduction

Polyethylene (PE) is widely used in agriculture and daily life due to its exceptional durability, low cost, and broad applicability (Purohit et al., 2025). However, PE is highly resistant to degradation under natural conditions (He et al., 2024; Kong et al., 2025). Its extensive use and improper disposal have resulted in serious “white pollution,” posing persistent environmental threats (Chen et al., 2024; Hossain et al., 2024). Therefore, developing efficient and eco-friendly technologies for PE degradation is urgently required. Among various treatment approaches, microbial degradation is considered one of the most promising green strategies because of its sustainability and environmental compatibility (Roager and Sonnenschein, 2019; Lin et al., 2022; Yang et al., 2025). Bacteria from more than 20 genera—such as *Pseudomonas* (Kim et al., 2025; Wanapat et al., 2025), *Bacillus* (Chen et al., 2025; Dhanraj et al., 2025), and *Acinetobacter* (Lyu et al., 2024)—have been reported to degrade PE (Gambarini et al., 2024). These microorganisms typically initiate the degradation process by forming biofilms on the PE surface, leading to subsequent physicochemical deterioration of the polymer (Amobonye et al., 2021; Chigwada and Tekere, 2023). Nevertheless, under most reported conditions, PE degradation by wild-type strains proceeds slowly and requires prolonged incubation, resulting in limited weight loss or minor chemical modification, which severely restricts their practical applicability in environmental remediation (Maroof et al., 2022; Tareen et al., 2022; Debbarma et al., 2024).

To address this limitation, mutational breeding—a classic and effective strategy for microbial strain improvement—has been widely employed to enhance desirable microbial traits. Random mutagenesis, in particular, offers advantages such as operational simplicity, low cost, and exemption from transgenic regulations. This approach involves exposing microorganisms to physical or chemical mutagens—such as ultraviolet radiation, microwave irradiation, or alkylating agents—to induce random genomic mutations (Hadad et al., 2005; Yu et al., 2020; Jo et al., 2022). Coupled with subsequent directional screening, it enables the selection of mutant strains with markedly enhanced performance. In recent years, this strategy has been increasingly applied to improve the degradation efficiency of recalcitrant polymers (Bhayana et al., 2022; Din et al., 2023; Hoffmam et al., 2023; Wang et al., 2025), highlighting its potential to overcome the intrinsic limitations of native PE-degrading bacteria. Indeed, multiple mutant strains with enhanced degradation capabilities have been successfully obtained through this approach, providing a reliable technical pathway for the development of highly efficient PE-degrading microorganisms (Ji et al., 2024; Xiong et al., 2025).

Members of the genus *Sphingobacterium* have been reported to degrade various recalcitrant compounds, including petroleum hydrocarbons, polycyclic aromatic hydrocarbons, lignin, and cellulose (Montazer et al., 2018; Kim et al., 2003; Bandopadhyay et al., 2020; Satti et al., 2021). These metabolic capabilities suggest that *Sphingobacterium* possesses a versatile enzymatic repertoire and a high degree of environmental adaptability, making it a promising yet underexplored chassis for polymer biodegradation (Van Beilen and Funhoff, 2007; Wasmund et al., 2009; Restrepo-Flórez et al., 2014; Shapiro et al., 2022; Ren et al., 2023). While previous studies have primarily focused on the intrinsic degradation potential of wild-type *Sphingobacterium* strains, their performance enhancement and adaptive plasticity through mutational breeding remain largely unexplored.

In this study, we applied optimized physical and chemical mutagenesis to *Sphingobacterium prati* BS2—a strain originally isolated from PE-mulched cotton field soil. Following directed screening with PE as the sole carbon source, we obtained several mutant strains and systematically evaluated their degradation performance and phenotypic stability. The results demonstrate that the combined mutagenesis approach effectively induced oxidative polymer deterioration and significantly elevated degradation performance. By linking mutagenesis-induced phenotypic variation with enhanced PE degradation, this work advances current understanding from descriptive biodegradation capability to inducible improvement at the mutational level. Altogether, this study expands the available microbial toolkit for PE degradation and contributes to the development of effective and sustainable bioremediation strategies.

## 2 Materials and methods

### 2.1 Bacterial strain, culture media, and polyethylene substrates

The PE-degrading bacterium *Sphingobacterium prati* BS2, previously isolated from cotton field soil containing residual plastic mulch, was used in this study. The wild-type strain was routinely cultured in Lysogeny Broth (LB) medium. All degradation assays were conducted in a defined mineral salt medium (MSM), the detailed composition of which is provided in Supplementary Information.

PE mulch film (approximately 1 × 1 cm strips) and powdered PE (150–200 μm particle size) were used as substrates. Prior to use, PE films were sequentially washed with 2% (w/v) sodium dodecyl sulfate (SDS) and 75% (v/v) ethanol, followed by UV sterilization for 30 min on each side. PE powder was sterilized by prolonged UV exposure and ethanol treatment to ensure the removal of potential contaminating microorganisms. Sterility was confirmed by incubating treated PE in LB medium and observing no microbial growth prior to degradation assays.

### 2.2 Mutagenesis and screening of mutants

Mutagenesis was performed on mid-exponential-phase cultures (17–24 h) of BS2 in LB medium. Both physical (ultraviolet irradiation, UV; microwave, MW) and chemical (acridine orange, AO; hydroxylamine hydrochloride, HY) mutagens were applied. For MW mutagenesis, ice-bath cooling was applied to reduce thermal stress, and the temperature of cultures was monitored to ensure uniform treatment (maintained at 0–4°C). Gradient tests were conducted to determine lethal doses, and treatments resulting in a lethality rate of 80–95% were selected for large-scale mutant generation. Details for each mutagen are provided in Supplementary Information. The lethality rate was calculated as:


Lethality(%)=[1-(Survivalcountaftertreatment/Survivalcountofcontrol)]×100. (1)

Mutants were primarily screened on MSM agar plates containing 1% (w/v) sterile PE powder as the sole carbon source. Colonies surrounded by a clear halo after 7–10 days of incubation at 30°C were considered positive. The correlation between halo zone diameter and actual PE weight loss was verified in preliminary tests. The positive mutation rate was calculated as:


PositiveMutationRate(%)=(Numberofpositivemutants/Totalnumberofsurvivors)×100. (2)

The most promising mutant from each treatment was designated according to the mutagenesis method (e.g., BS2-UV from UV mutagenesis). A synergistic mutant, BS2-UA, was generated by first subjecting mid-exponential-phase BS2 cultures to UV irradiation under the optimized conditions (40 s, 88.85% lethality) in liquid, followed immediately by AO treatment at 0.045% for 24 h on plates, with the surviving cells subsequently recovered and screened for enhanced PE degradation.

### 2.3 Stability assessment and degradation performance

Selected mutants serially subcultured on PE-MSM plates for 20 successive generations. The diameter of the clear (degradation) halo was measured every generation to evaluate phenotypic stability, expressed as the coefficient of variation (CV). All stability assays were performed in MSM with PE as the sole carbon source to ensure relevance to degradation capacity.

For degradation assays, a single colony was inoculated into 20 mL of MSM in a 50 mL Erlenmeyer flask containing a pre-weighed PE film strip. Flasks were incubated at 30°C with shaking at 150 rpm for 50 days. Bacterial growth was monitored by measuring optical density at 600 nm (OD_600_), and the culture pH was recorded periodically.

Biofilm formation on the PE film was quantified using the crystal violet staining method. Briefly, after incubation, films were gently washed, stained with 0.1% crystal violet, destained with ethanol, and the absorbance of the solubilized dye was measured at 570 nm.

### 2.4 Analysis of PE degradation

*Gravimetric Analysis:* After cultivation, PE films were recovered, thoroughly washed with 2% SDS and distilled water to remove attached biomass, and dried to constant weight in a vacuum desiccator. The weight loss percentage was calculated.

*Surface Morphology:* Washed and dried PE films were sputter-coated with gold and observed using a scanning electron microscope (SEM; SU8010, Hitachi). SEM sample preparation was carefully controlled to avoid introducing artifacts.

*Chemical Structure Analysis:* Chemical changes were analyzed by attenuated total reflectance Fourier-transform infrared spectroscopy (ATR-FTIR; Vertex 70v, Bruker). Spectra were collected in the range of 4,000–500 cm^–1^. The hydroxyl index (HI) and carbonyl index (CI) were calculated as the ratio of the integrated absorbance of the ∼3,400 and ∼1,715 cm^–1^ regions, respectively, to that of the reference band at ∼1,465 cm^–1^ (CH2 bending).

### 2.5 Statistical analysis

All experiments were performed with at least three biological replicates. Data are presented as mean ± standard deviation. Significant differences among groups were determined by one-way analysis of variance (ANOVA) followed by the least significant difference (LSD) *post-hoc* test using the R statistical environment. A *P* < 0.05 was considered statistically significant.

Detailed experimental procedures, including mutagenesis parameters, screening protocols, culture conditions, and analytical methods, are provided in Supplementary Information.

## 3 Results

### 3.1 Mutagenesis optimization and mutant isolation

To ensure reliable mutagenesis outcomes, cultures were selected during the late exponential to early stationary phase. In LB liquid medium, the wild-type *Sphingobacterium prati* BS2 exhibited a lag phase from 0 to 5 h, an exponential growth phase between 5 and 17 h, and reached the stationary phase around 24 h (Supplementary Figure 1). Cultures aged 17–24 h were therefore selected for subsequent mutagenesis experiments on BS2.

**FIGURE 1:**
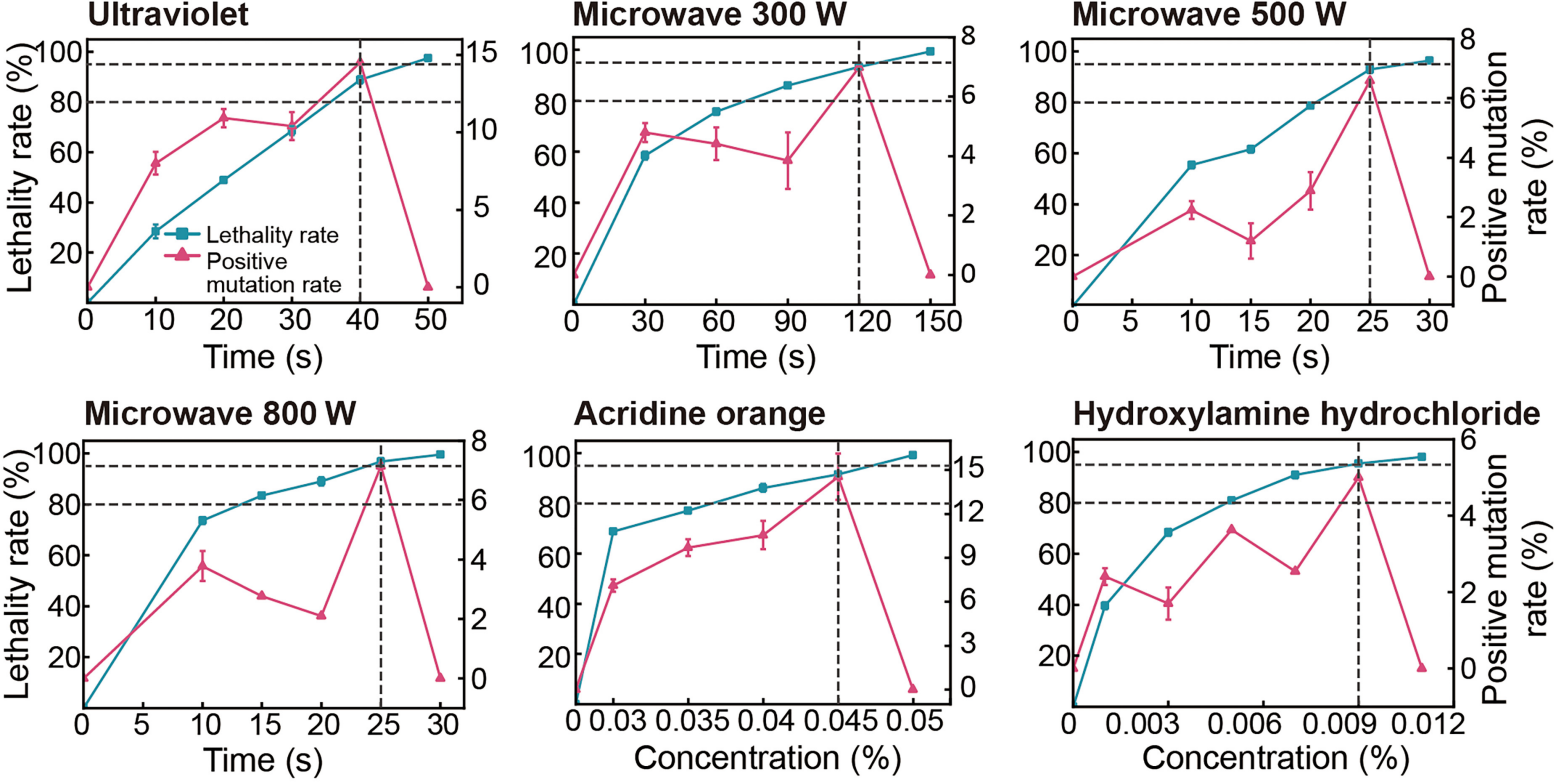
Optimization of mutagenesis conditions for strain BS2.

Lethality and positive mutation rates reveal an optimal window for mutagenesis (Figure 1). BS2 lethality increased with treatment intensity, and the positive mutation rate exhibited a clear peak within a narrow lethality window of 80–95% before declining sharply, within which mutant recovery is maximized. Based on these observations, the optimal mutagenesis conditions were established as follows: UV irradiation for 40 s (positive mutation rate 14.52%, lethality 88.85%), microwave (MW) at 300 W for 120 s (6.98%, 93.42%), 500 W for 25 s (6.59%, 93.04%), 800 W for 25 s (7.14%, 96.79%), acridine orange (AO) at 0.045% (14.55%, 91.58%), and hydroxylamine hydrochloride (HY) at 0.009% (5.00%, 95.41%). All selected conditions fell within the lethality range associated with higher positive mutation rates, facilitating the screening of mutants with enhanced PE-degrading capacity.

The resulting strains were designated according to the mutagenesis treatment: wild-type BS2, BS2-UV, BS2-300W, BS2-500W, BS2-800W, BS2-AO, and BS2-HY.

The rate of positive mutation under PE stress peaks at lethality rates between 80 and 95% across treatments. Data are presented as mean ± SD (*n* = 3). Two horizontal dashed lines indicate a lethality of 80–95%, and the vertical dashed line indicates the highest positive mutation rate. Mutagenesis details are provided in Supplementary Information.

### 3.2 Mutant strains exhibit enhanced and stable PE-degrading phenotypes

**FIGURE 2:**
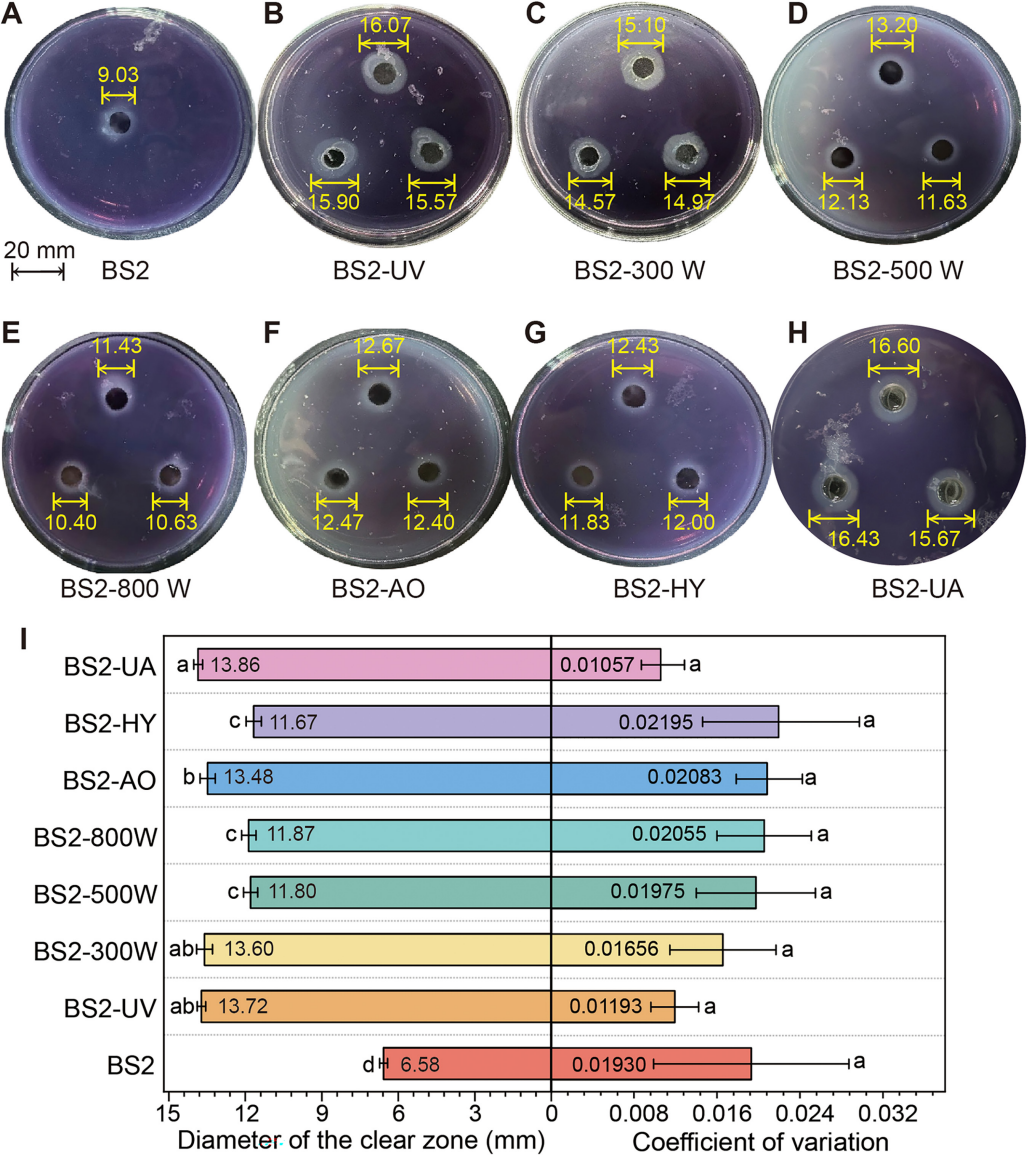
Stability of Halo zone formation over 20 successive generations. **(A–H)** Representative halo zones produced by the test strains at the first generation. The indicated values are zone diameters (mm). **(I)** Stability assessment: Halo zone diameter and CV over 20 successive generations. Data are presented as mean ± SD (*n* = 3 biological replicates for halo zone diameter; *n* = 20 generations for CV), with mean values displayed inside the bars. Different letters indicate significant differences among strains (*P* < 0.05, one-way ANOVA with LSD *post-hoc* test).

Clear-zone–based screening revealed pronounced differences in PE-degrading capacity among mutants derived from different mutagenic treatments (Figure 2). After 20 successive subcultures, the diameters of clear zones—indicative of PE degradation—on PE-containing medium were in the following order: BS2-UA > BS2-UV > BS2-300W > BS2-500W > BS2-800W > BS2-AO > BS2-HY (Figure 2I). Notably, BS2-UA, a synergistic mutant generated by sequential UV and AO treatments with a moderate lethality rate of 81.93 ± 3.41% but the highest positive mutation rate of 23.70 ± 3.40%, exhibited the largest clear zones and highest phenotypic stability (CV = 0.01), making it the most promising strain for PE degradation.

Phenotypic stability analysis further demonstrated that the enhanced degradation traits were robust across generations (Figure 2I). All mutant strains showed CV values below 0.1 in halo zone diameter over 20 successive generations on PE-based selective medium, indicating high intergenerational stability of PE-degrading performance. Accordingly, based on a combined assessment of degradation capacity (halo zone diameter) and phenotypic stability (CV), strains BS2-UA, BS2-UV, BS2-300W, and BS2-AO were selected for subsequent degradation performance analyses.

### 3.3 Enhanced biomass accumulation, acidification, biofilm formation, and polyethylene degradation in mutant strains

**FIGURE 3:**
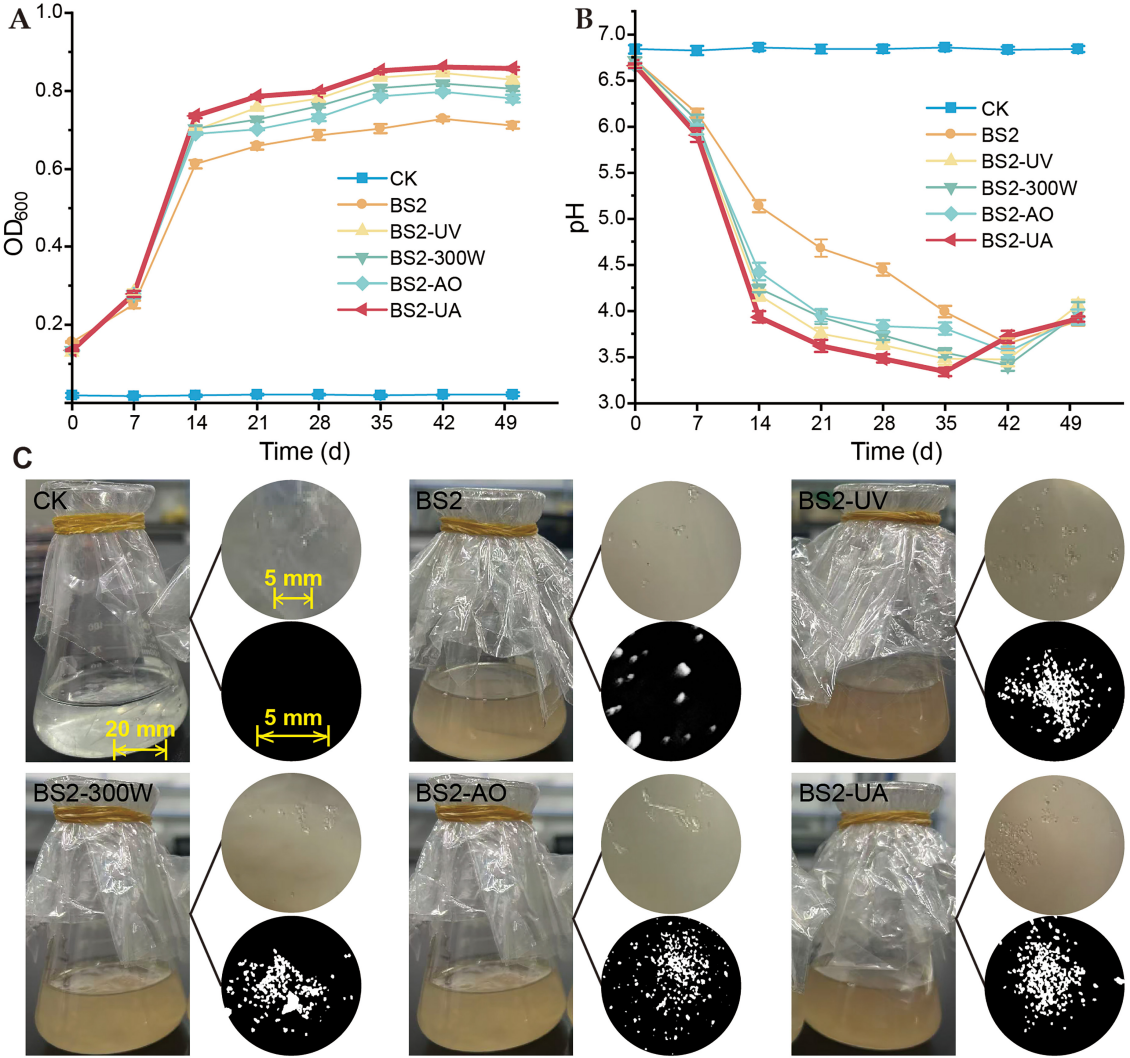
Planktonic growth, medium acidification, and physical fragmentation of polyethylene (PE) during biodegradation by wild-type and mutant strains. **(A)** Temporal changes in planktonic cell density (OD600) of different strains cultured in mineral salt medium with PE film as the sole carbon source over a 50-day incubation period. **(B)** Changes in culture pH during incubation illustrate distinct acidification patterns among the mutant strains, with BS2-UA showing a rapid decrease to a lower pH followed by a gradual recovery. **(C)** Macroscopic evidence of PE fragmentation after 50 days of incubation. Left: representative photograph of a degradation culture; upper right inset: surface deterioration of the PE film; lower right inset: fine PE debris recovered by filtration, indicating extensive physical destabilization of the polymer. Data are presented as mean ± SD (*n* = 3). CK, uninoculated control; BS2, wild-type *Sphingobacterium prati* BS2; UV, ultraviolet light (40 s); MW, microwave (300 W for 120 s); AO, acridine orange (0.045%); HY, hydroxylamine hydrochloride (0.009%); UA, sequential UV + AO mutagenesis.

In PE-only medium, all mutant strains exhibited enhanced planktonic growth compared with the wild-type BS2, as reflected by higher final OD_600_ values over the 50-day incubation period (Figure 3A). Following an initial rapid increase during the first 15–20 days, biomass accumulation gradually plateaued, suggesting that growth became limited by substrate availability or accumulation of inhibitory metabolites.

Concomitant with biomass accumulation, the culture pH of all inoculated treatments declined markedly from an initial value of ∼6.8 to approximately 3.3–4.0, whereas the uninoculated control (CK) remained stable throughout the incubation (Figure 3B). Among the inoculated treatments, BS2-UA exhibited one of the most rapid and pronounced acidification responses, with a sharp pH decrease occurring within the first 2 weeks and the lowest pH reached around day 35. In contrast, the other mutant strains showed relatively delayed acidification, reaching their minimum pH between days 35 and 42. Although BS2-UA began to recover slightly earlier, its pH remained among the lowest during the mid-to-late incubation period, indicating a deeper acidification phase. The other strains, however, displayed a more rapid pH increase after reaching their minima, reflecting a comparatively shorter duration at peak acidification. These results reveal distinct acidification dynamics among the mutant strains, with BS2-UA characterized by a rapid onset, deeper acidification, and gradual recovery.

Macroscopic inspection of the degradation cultures revealed clear differences in the physical integrity of PE films among treatments (Figure 3C). While the uninoculated control retained intact and transparent PE films, cultures inoculated with mutant strains displayed visible PE fragmentation, increased turbidity, and accumulation of fine debris suspended in the medium. Among the mutants, BS2-UA and BS2-UV exhibited the most pronounced physical deterioration, with a higher apparent abundance of film fragments and broader distribution of sizes compared with other strains. These observations indicate enhanced physical destabilization of the polymer matrix by the mutants.

**FIGURE 4:**
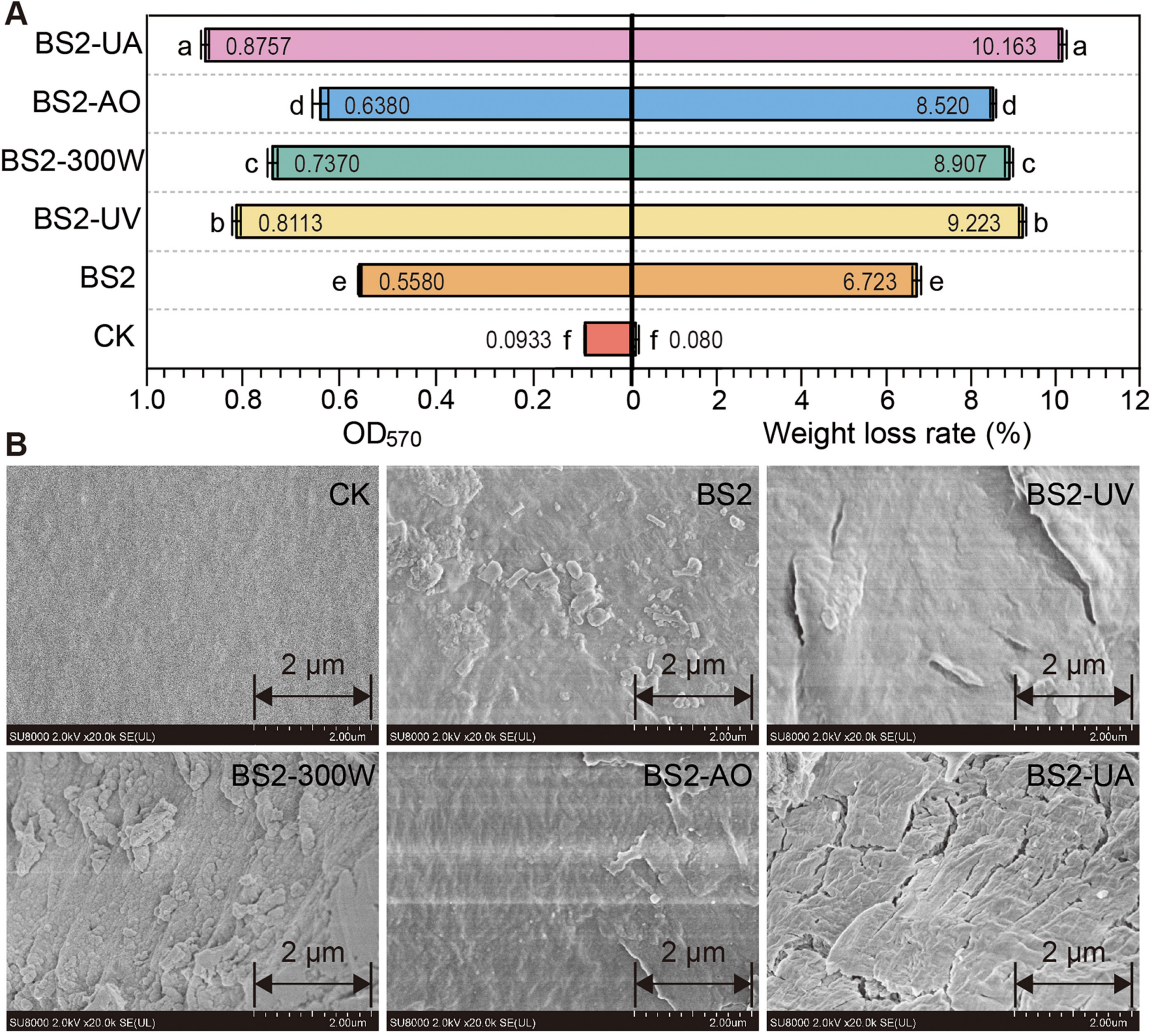
Polyethylene (PE) biodegradation efficiency and corresponding changes in surface morphology. **(A)** Biofilm biomass on PE film surfaces (crystal violet assay, left) and PE weight loss rate (right). Data are mean ± SD (*n* = 3), with mean values displayed inside the bars. Different letters indicate significant differences (*P* < 0.05, one-way ANOVA with LSD *post-hoc* test). **(B)** Scanning electron microscopy (SEM) images of PE surfaces after degradation. Mutant-treated films displayed pronounced wrinkles, cracks, and erosion features, in contrast to the smooth surface of the uninoculated control. scale bar = 2 μm. CK, uninoculated control; BS2, wild-type *Sphingobacterium prati* BS2; UV, ultraviolet light (40 s); MW, microwave (300 W for 120 s); AO, acridine orange (0.045%); HY, hydroxylamine hydrochloride (0.009%); UA, UV + AO treatment.

Quantitative assessment of biofilm biomass using the crystal violet assay revealed that all mutant strains formed significantly more biofilm than the wild-type BS2 (Figure 4A, left). BS2-UA exhibited the highest biofilm accumulation (56.9% increase over BS2), followed by BS2-UV (45.4%), BS2-300W (32.1%), and BS2-AO (14.3%), suggesting that mutagenesis enhanced the ability of the strains to colonize and attach to the PE surface.

Consistent with these observations, all mutant strains exhibited significantly higher PE weight loss compared with BS2 (Figure 4A, right). BS2-UA achieved the highest degradation efficiency (10.16 ± 0.22%), representing a 51.17% increase over the wild-type strain (6.72 ± 0.18%), followed by BS2-UV (9.22 ± 0.19%, + 37.18%), BS2-300W (8.90 ± 0.21%, + 32.47%), and BS2-AO (8.51 ± 0.20%, + 26.72%).

Scanning electron microscopy (SEM) further corroborated the gravimetric data, revealing extensive surface deterioration, including pronounced wrinkles, cracks, and erosion features on PE films incubated with mutant strains, particularly BS2-UA and BS2-UV, in contrast to the smooth surface observed in the control (Figure 4B). The severity of surface damage closely paralleled biofilm biomass and PE weight loss.

### 3.4 FTIR analysis reveals enhanced oxidative modification of polyethylene

**FIGURE 5:**
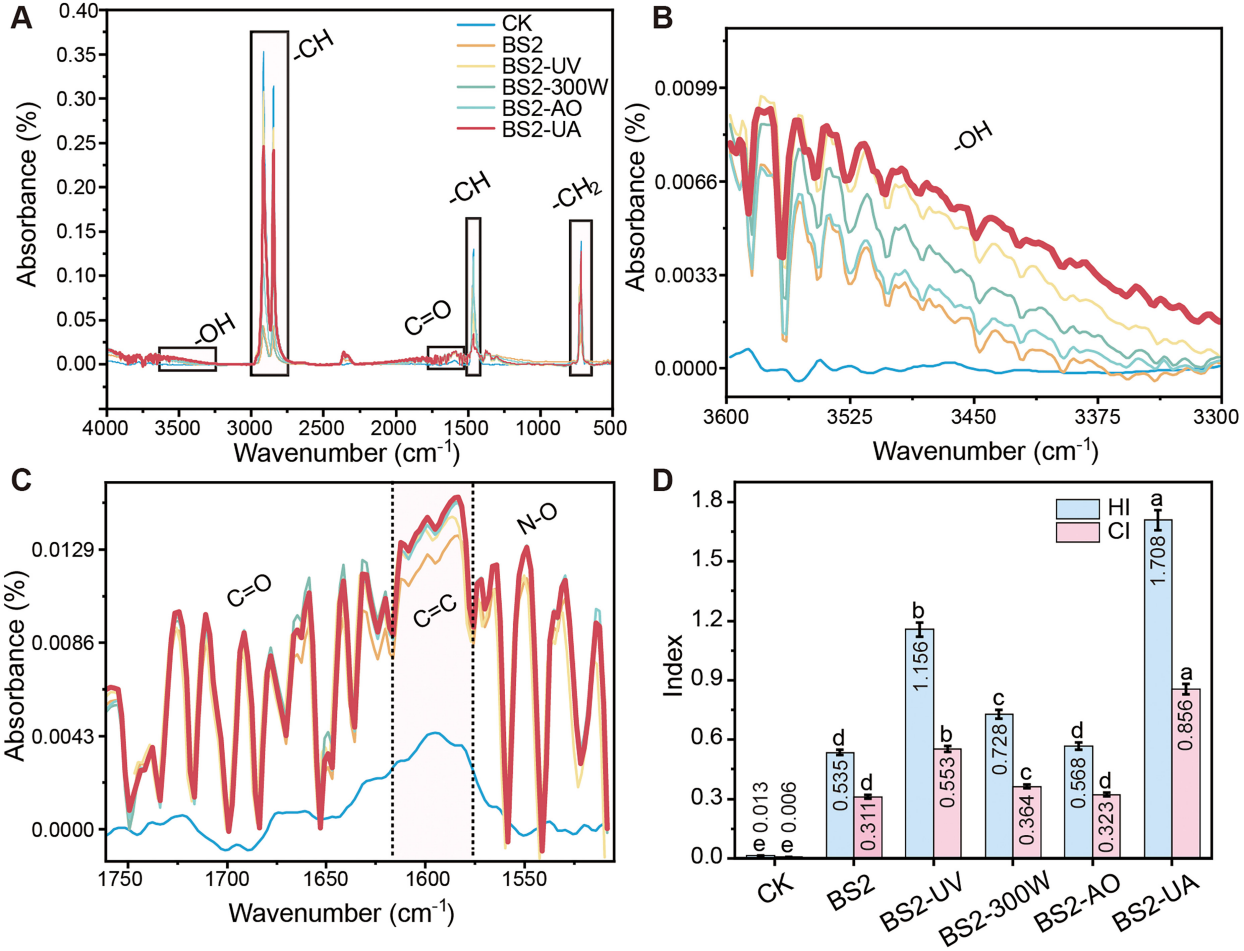
Chemical structural alterations in polyethylene (PE) after biodegradation. **(A)** Full ATR-FTIR (attenuated total reflectance Fourier-transform infrared) spectra of PE after incubation with the test strains. Key chemical groups are indicated, including –OH (∼3,400cm^–1^), C–H stretching/bending (2,912–2,920 and 2,848–2,850cm^–1^), C = O (∼1,715cm^–1^), and C–O (∼1,100–1,300 cm^–1^). **(B)** Enlarged hydroxyl (–OH) stretching region. **(C)** Enlarged oxygen-containing functional group region (C = O, C–O). **(D)** Hydroxyl index (HI, A∼3,400 cm^–1^/A∼1,465 cm^–1^) and carbonyl index (CI, A∼1,715 cm^–1^/A∼1,465 cm^–1^) of PE after treatments. Increased HI and CI values correspond to greater oxidation and chain scission of the polymer. Data are presented as mean ± SD (*n* = 3), with mean values displayed inside the bars for clarity. Different letters indicate significant differences among each index (HI or CI) (*P* < 0.05, one-way ANOVA with LSD *post-hoc* test). CK, uninoculated control; BS2, wild-type *Sphingobacterium prati* BS2; UV, ultraviolet light (40 s); MW, microwave (300 W for 120 s); AO, acridine orange (0.045%); HY, hydroxylamine hydrochloride (0.009%); UA, UV + AO treatment.

FTIR analysis further revealed that incubation with BS2 mutants induced pronounced chemical modifications of PE (Figure 5), consistent with the macroscopic fragmentation and SEM-observed surface deterioration. Characteristic C–H stretching and bending peaks at 2,912–2,918 cm^–1^ and 2,848–2,850 cm^–1^ were attenuated and slightly shifted (Figure 5A), indicating scission of polymer chains. Simultaneously, new absorption bands corresponding to hydroxyl (–OH) (Figure 5B), carbonyl (C = O), and ether (C–O) groups emerged (Figure 5C), with intensities relatively higher in mutant-treated samples than in those incubated with the wild-type strain.

Quantitative analysis of the hydroxyl index (HI, A∼3,400 cm^–1^/A∼1,465 cm ^–1^) and carbonyl index (CI, A∼1,715 cm ^–1^/A∼1,465 cm ^–1^) was consistent with these observations, with the highest index values observed for BS2-UA, followed by BS2-UV and BS2-300W (Figure 5D). BS2-UA achieved HI and CI values of 1.71 and 0.86, representing 2.51- and 1.75-fold increases over the wild-type, respectively. These results paralleled the previously observed metabolic acidification and the increased biofilm formation observed for BS2-UA.

Together, these observations support enhanced PE degradation in the mutagenesis-derived strains, with BS2-UA showing the most pronounced chemical and physical modifications, consistent with its superior degradation performance.

## 4 Discussion

The development of microbial resources for PE degradation has attracted increasing attention due to the recalcitrant nature of this polymer. However, the degradation efficiency of most environmental strains remains low, and systematic strategies to enhance microbial PE-degrading capacity are still limited. In this study, we applied physical, chemical, and combined mutagenesis approaches to a laboratory-preserved strain, *Sphingobacterium prati* BS2, originally isolated from plastic-mulched soil, to improve its degradation performance. Several superior mutants (BS2-UV, BS2-300W, BS2-AO, and BS2-UA) were obtained, exhibiting enhanced PE degradation, as evidenced by increased biofilm formation, higher PE weight loss, and elevated hydroxyl and carbonyl indices (HI and CI). These findings demonstrate that mutational breeding can effectively enhance the PE-degrading capacity of environmental bacteria, providing a foundation for further microbial plastic biodegradation research.

### 4.1 Variation in mutagenesis efficiency

The wild-type strain BS2 displayed metabolic activity under PE stress, highlighting the physiological robustness of *Sphingobacterium*. Members of this genus can utilize complex carbon sources, including hydrocarbons and lignin (Montazer et al., 2018; Kim et al., 2003; Bandopadhyay et al., 2020; Satti et al., 2021), suggesting an intrinsic metabolic potential for attacking recalcitrant polymers. This capability is likely associated with specific enzymatic systems, such as alkane monooxygenases (AlkB and Cyp153) (Van Beilen and Funhoff, 2007; Wasmund et al., 2009; Shapiro et al., 2022) and lignin- and manganese-dependent peroxidases (Ren et al., 2023), which are functionally relevant to PE degradation given the structural similarities between PE, alkanes, and lignin (Restrepo-Flórez et al., 2014; Jeon and Kim, 2015). In addition, biosurfactant production may facilitate bacterial attachment and biofilm formation on hydrophobic PE surfaces, creating a favorable microinterface for sustained degradation (Marqués et al., 2012). Building on these traits, the present study demonstrates that *Sphingobacterium*’s inherent degradative potential can be further amplified and stabilized through targeted mutagenesis.

BS2 showed distinct responses to different mutagenesis. UV-derived mutants exhibited higher positive mutation rates and larger halo zones than MW-derived mutants, likely due to UV-induced DNA damage, including random base substitutions and strand breaks, which increase mutation diversity (Ilmjärv et al., 2017; Song et al., 2025). This finding aligns with previous reports on UV mutagenesis of hydrocarbon-degrading strains, suggesting that UV is particularly effective for developing polymer-degrading microorganisms (Xia et al., 2017).

In contrast, BS2 showed relatively low tolerance to MW mutagenesis. While 300 W treatment produced larger halo zones than other MW settings, overall efficiency remained limited. Moderate MW may enhance mutation frequency, but excessive power causes thermal damage, denaturing proteins and harming cells (Jangid et al., 2010; Banerjee et al., 2013). Even with ice-bath cooling, thermal interference could not be completely eliminated (Zutterling et al., 2018; Joseph and Badrinarayanan, 2020).

Chemical mutagenesis also yielded distinct effects. Acridine orange (AO)-derived mutants showed higher positive mutation rates and larger halo zones than hydroxylamine (HY)-derived mutants, consistent with AO’s mechanism of intercalating into DNA and disrupting replication, whereas HY induces specific nucleotide modifications (Phillips and Brown, 1967; Ayling and Snell, 1968).

Beyond the specific mechanisms, mutagenesis not only enhanced traits directly related to PE degradation but also promoted broader system-level adaptive capacities under PE stress, including sustained metabolic activity and environmental modification (Yang et al., 2024; Huang et al., 2025; Najar-Almanzor et al., 2025).

### 4.2 Integrated metabolic activity, acidification dynamics, and the role of synergistic mutagenesis in BS2-UA

Among the mutant strains, BS2-UA exhibited the highest PE degradation and the most integrated expression of degradation-associated traits, achieving a weight loss of 10.16% under our experimental conditions. This performance falls within the upper range of previously reported PE-degrading bacteria, such as *Pseudomonas aeruginosa* (2.66%) (Wanapat et al., 2025), *Exiguobacterium* sp. (5.70%) (Maroof et al., 2022), and *Brevundimonas naejangsanensis* (4.64%) (Singh et al., 2023), although direct comparison across studies should be interpreted cautiously due to differences in experimental conditions. The enhanced degradation efficiency of BS2-UA likely reflects its inherent metabolic versatility and adaptive traits.

The distinct temporal pH profile of BS2-UA reveals its enhanced metabolic activity and coordinated physiological response during PE degradation. Compared with other strains, BS2-UA exhibited a more rapid and pronounced decline in pH during the early degradation stage, reaching its minimum around day 35. This accelerated acidification indicates intensified metabolic flux and elevated production of acidic intermediates associated with oxidative PE modification, while metabolic activity is sustained through self-regulatory mechanisms (Ojha et al., 2017; Hou et al., 2019; Fang et al., 2024; Tiwari et al., 2024).

This strong and sustained acidification coincided with multiple superior performance indicators of BS2-UA, including higher biomass accumulation under PE stress, enhanced biofilm formation, and the highest PE weight loss among all tested strains. The prolonged low-pH environment suggests sustained metabolic activity rather than a transient stress response, promoting continuous surface oxidation and chain scission. Simultaneously, the dense biofilm matrix enhances cell–surface contact and stabilizes localized reaction conditions via extracellular proteins and polysaccharides, strengthening adhesion to the hydrophobic PE surface and maintaining microenvironments favorable for degradation reactions (Dey et al., 2020; Wang et al., 2021).

In contrast, other mutant strains reached their minimum pH later and showed a faster rebound, consistent with lower biomass accumulation and weaker degradation performance. These differences highlight that effective PE degradation depends not only on the capacity to acidify the surrounding environment, but also on the temporal coordination between metabolic intensity, physiological stability, and environmental modification—particularly pronounced in BS2-UA.

During the late degradation stage, BS2-UA exhibited a gradual, controlled recovery of pH. This moderated rebound likely reflects reutilization or further metabolism of acidic intermediates, indicating a more balanced and resilient metabolic network. Such regulation may allow BS2-UA to maintain prolonged activity while avoiding excessive self-inhibition under acidic conditions.

These integrated metabolic and physiological advantages align with the synergistic UV–AO mutagenesis strategy employed to generate BS2-UA. Unlike single treatments, synergistic mutagenesis may induce complementary genetic alterations affecting core metabolic and regulatory networks, including stress response, biofilm formation, and metabolic flux control (Han et al., 2020; Zhang et al., 2022). UV generates initial genetic diversity via DNA strand breaks and base damage, while AO further expands and stabilizes variation by modulating DNA replication and repair (Nakai and Saeki, 1964; Barker and Hardman, 1978; Mao et al., 2015; Lu et al., 2022; Yu et al., 2024; Zhao et al., 2024). Rather than simply increasing mutation load, combined UV–AO treatment likely favored selection of mutants with coordinated and stable phenotypic traits—a hypothesis warranting future genomic investigation. Consequently, combined mutagenesis proved particularly effective for improving polymer-degrading microorganisms, extending prior applications from hydrocarbon- and surfactant-degrading strains to PE (Han et al., 2020; Zhang et al., 2022).

Consistent with this interpretation, surface morphology and FTIR analyses revealed more pronounced structural damage and higher enrichment of oxygen-containing functional groups (–OH and C = O) in PE treated with BS2-UA, indicating effective chemical modification of the polymer chains (Restrepo-Flórez et al., 2014; Jiang et al., 2023). Taken together, the convergence of rapid early acidification, sustained metabolic activity, robust biofilm formation, and enhanced oxidative modification supports the conclusion that synergistic mutagenesis confers a systems-level advantage, underpinning the superior PE degradation performance of BS2-UA.

Although conducted under controlled laboratory conditions with PE as the sole carbon source, the results demonstrate that enhanced environmental adaptability—manifested as increased biomass accumulation, biofilm formation, and sustained metabolic activity—is a key driver of improved PE degradation in the mutant strains. These traits are likely critical determinants of strain performance in more complex environments. Future studies using soil or compost microcosms are necessary to evaluate whether these adaptability advantages translate into effective colonization, competitiveness with indigenous microbial communities, and sustained PE degradation under environmentally realistic conditions.

## 5 Conclusion

Targeted mutagenesis of *Sphingobacterium prati* BS2 using physical, chemical, and combined UV–AO treatments yielded superior mutants with markedly enhanced PE degradation. The mutants, particularly BS2-UA, exhibited coordinated improvements in biomass accumulation, biofilm formation, culture acidification, PE weight loss, and incorporation of oxygen-containing functional groups. The outstanding performance of BS2-UA is best explained by the integrated expression of multiple advantageous traits, including sustained metabolic activity and a characteristic acidification pattern, rather than a single dominant mechanism, consistent with the effects of synergistic mutagenesis in promoting coordinated and sustained phenotypic responses. In this context, the combined UV–AO strategy appears to facilitate the selection of mutants with enhanced metabolic activity, stable surface colonization, and effective environmental modification, collectively supporting efficient PE degradation. Overall, these findings demonstrate that synergistic mutagenesis represents an effective strategy for enhancing the latent biodegradation potential of underexplored environmental bacteria, providing not only high-performance PE-degrading candidates but also valuable methodological insights for the development of microbial resources targeting recalcitrant plastic polymers.

## Data Availability

The original contributions presented in this study are included in the article/Supplementary material, further inquiries can be directed to this corresponding authors.

## References

[B1] Amobonye A. Bhagwat P. Singh S. Pillai S. (2021). Plastic biodegradation: frontline microbes and their enzymes. *Sci. Total Environ.* 759:143536. 10.1016/j.scitotenv.2020.143536 33190901

[B2] Ayling J. E. Snell E. E. (1968). Relation of structure to activity of pyridoxal analogs as substrates for pyridoxamine-pyruvate transaminase. *Biochemistry* 7 1626–1636. 10.1021/bi00845a003 5650371

[B3] Bandopadhyay S. Liquet Y. González J. E. Henderson K. B. Anunciado M. B. Hayes D. G. et al.. (2020). Soil microbial communities associated with biodegradable plastic mulch films. *Front. Microbiol.* 11:587074. 10.3389/fmicb.2020.587074 33281783 PMC7691482

[B4] Banerjee B. Ahmed R. Chandna S. Abegaonkar M. Tripathi A. Deshmukh P. et al.. (2013). Detection of low level microwave radiation induced deoxyribonucleic acid damage vis-à-vis genotoxicity in brain of fischer rats. *Toxicol. Int.* 20:19. 10.4103/0971-6580.111549 23833433 PMC3702122

[B5] Barker G. R. Hardman N. (1978). The effects of acridine orange on deoxyribonucleic acid in *Escherichia coli*. *Biochem. J.* 171 567–573. 10.1042/bj1710567 27167 PMC1184001

[B6] Bhayana T. Saxena A. Gupta S. Dubey A. K. (2022). Enhanced decolourisation and degradation of azo dyes using wild versus mutagenic improved bacterial strain: a review. *Vegetos* 36 28–37. 10.1007/s42535-022-00496-y

[B7] Chen S.-Y. Noor S. Li Z. Zhao Z.-H. Li C.-H. (2024). Tough, recyclable and degradable plastics with multiple functions based on supramolecular covalent adaptive networks. *J. Mater. Chem. A* 12 21321–21333. 10.1039/D4TA02644F

[B8] Chen X. Xue H. Jiang Z. Zhao J. Xu T. Su J. et al.. (2025). Biodegradation of polyethylene by Gordonia sp. C1 and Bacillus sp. C2 isolated from landfill. *J. Environ. Chem. Eng.* 13:116443. 10.1016/j.jece.2025.116443

[B9] Chigwada A. D. Tekere M. (2023). The plastic and microplastic waste menace and bacterial biodegradation for sustainable environmental clean-up a review. *Environ. Res.* 231:116110. 10.1016/j.envres.2023.116110 37172684

[B10] Debbarma P. Suyal D. C. Kumar S. Zaidi M. G. H. Goel R. (2024). Comparative e-waste plastics biodegradation efficacy of monoculture *Pseudomonas aeruginosa* strain PE10 and bacterial consortium under in situ condition. *Front. Microbiol.* 14:1277186. 10.3389/fmicb.2023.1277186 38304861 PMC10830738

[B11] Dey A. S. Bose H. Mohapatra B. Sar P. (2020). Biodegradation of Unpretreated Low-Density Polyethylene (LDPE) by *Stenotrophomonas* sp. and Achromobacter sp., isolated from waste dumpsite and drilling fluid. *Front. Microbiol.* 11:603210. 10.3389/fmicb.2020.603210 33391224 PMC7775675

[B12] Dhanraj N. D. Sreelakshmi U. P. Sneha P. Jisha M. S. (2025). A mechanistic insight into polyethylene degradation by Bacillus sp. and, Lysinibacillus sp. from mangrove soil. *Process Biochem.* 153 294–303. 10.1016/j.procbio.2025.03.018

[B13] Din A. Qadri Z. A. Wani M. A. Iqbal S. Malik S. A. Zargar S. M. et al.. (2023). Comparative analysis of physical and chemical mutagenesis in chrysanthemum cv. ‘Candid’: assessing genetic variation and breeding potential. *ACS Omega* 8 43836–43849. 10.1021/acsomega.3c05723 38027373 PMC10666220

[B14] Fang X. Cai Z. Wang X. Liu Z. Lin Y. Li M. et al.. (2024). Isolation and identification of four strains of bacteria with potential to biodegrade polyethylene and polypropylene from mangrove. *Microorganisms* 12:2005. 10.3390/microorganisms12102005 39458314 PMC11509307

[B15] Gambarini V. Drost C. J. Kingsbury J. M. Weaver L. Pantos O. Handley K. M. et al.. (2024). Uncoupled: investigating the lack of correlation between the transcription of putative plastic-degrading genes in the global ocean microbiome and marine plastic pollution. *Environ. Microbiome* 19:34. 10.1186/s40793-024-00575-4 38750536 PMC11097532

[B16] Hadad D. Geresh S. Sivan A. (2005). Biodegradation of polyethylene by the thermophilic bacterium *Brevibacillus borstelensis*. *J. Appl. Microbiol.* 98 1093–1100. 10.1111/j.1365-2672.2005.02553.x 15836478

[B17] Han S.-F. Jin W. Tu R. Ding B. Zhou X. Gao S. et al.. (2020). Screening and mutagenesis of high-efficient degrading bacteria of linear alkylbenzene sulfonates. *Chemosphere* 245:125559. 10.1016/j.chemosphere.2019.125559 31841794

[B18] He Y. Deng X. Jiang L. Hao L. Shi Y. Lyu M. et al.. (2024). Current advances, challenges and strategies for enhancing the biodegradation of plastic waste. *Sci. Total Environ.* 906:167850. 10.1016/j.scitotenv.2023.167850 37844647

[B19] Hoffmam Z. B. Soares L. B. De Morais E. R. Souza J. M. De Andrade A. L. D. De Jesus C. D. F. et al.. (2023). Evolutionary engineering and chemical mutagenesis of *Propionibacterium acidipropionici* for improved propionic acid production from sugarcane-derived saccharides. *Process Biochem.* 130 584–594. 10.1016/j.procbio.2023.05.022

[B20] Hossain M. M. Banerjee A. Chatterjee M. Roy K. Cronin M. T. D. (2024). QSPR and q-RASPR predictions of the adsorption capacity of polyethylene, polypropylene and polystyrene microplastics for various organic pollutants in diverse aqueous environments. *Environ. Sci.* 11 4196–4210. 10.1039/D4EN00311J

[B21] Hou L. Xi J. Chen X. Li X. Ma W. Lu J. et al.. (2019). Biodegradability and ecological impacts of polyethylene-based mulching film at agricultural environment. *J. Hazardous Mater.* 378:120774. 10.1016/j.jhazmat.2019.120774 31226592

[B22] Huang T. Cao S. Li X. Wang C. Peng X. (2025). Induced mutagenesis and comparative genomics of Raoultella sp. 64 for enhanced antimony resistance and biosorption. *Microorganisms* 13:880. 10.3390/microorganisms13040880 40284716 PMC12029485

[B23] Ilmjärv T. Naanuri E. Kivisaar M. (2017). Contribution of increased mutagenesis to the evolution of pollutants-degrading indigenous bacteria. *PLoS One* 12:e0182484. 10.1371/journal.pone.0182484 28777807 PMC5544203

[B24] Jangid R. K. Sharma R. Sudarsan Y. Eapen S. Singh G. Purohit A. K. (2010). Microwave treatment induced mutations and altered gene expression in *Vigna aconitifolia*. *Biologia Plant* 54 703–706. 10.1007/s10535-010-0124-x

[B25] Jeon H. J. Kim M. N. (2015). Functional analysis of alkane hydroxylase system derived from *Pseudomonas aeruginosa* E7 for low molecular weight polyethylene biodegradation. *Int. Biodeterioration Biodegrad.* 103 141–146. 10.1016/j.ibiod.2015.04.024

[B26] Ji S. H. Yoo S. Park S. Lee M. J. (2024). Biodegradation of low-density polyethylene by plasma-activated Bacillus strain. *Chemosphere* 349:140763. 10.1016/j.chemosphere.2023.140763 38029935

[B27] Jiang R. Lu G. Dang T. Wang M. Liu J. Yan Z. et al.. (2023). Insight into the degradation process of functional groups modified polystyrene microplastics with dissolvable BiOBr-OH semiconductor-organic framework. *Chem. Eng. J.* 470:144401. 10.1016/j.cej.2023.144401

[B28] Jo H.-G. Adidjaja J. J. Kim D.-K. Park B.-S. Lee N. Cho B.-K. et al.. (2022). Comparative genomic analysis of Streptomyces rapamycinicus NRRL 5491 and its mutant overproducing rapamycin. *Sci. Rep.* 12:10302. 10.1038/s41598-022-14199-6 35717543 PMC9206652

[B29] Joseph A. M. Badrinarayanan A. (2020). Visualizing mutagenic repair: novel insights into bacterial translesion synthesis. *FEMS Microbiol. Rev.* 44 572–582. 10.1093/femsre/fuaa023 32556198 PMC7476773

[B30] Kim B. C. Sohn C. K. Lim S. K. Lee J. W. Park W. (2003). Degradation of polyvinyl alcohol by Sphingomonas sp. SA3 and its symbiote. *J. Ind. Microbiol. Biotechnol.* 30 70–74. 10.1007/s10295-002-0010-4 12545389

[B31] Kim Y.-B. Cheon S. Yun S.-D. Kim S. Kim H. G. Seo M.-J. et al.. (2025). Genomic and enzymatic analysis of polyethylene biodegradation by *Pseudomonas fluorescens* JNU01 isolated from landfill environments. *J. Hazardous Mater. Adv.* 19:100857. 10.1016/j.hazadv.2025.100857

[B32] Kong Y. Wang R. Zhou Q. Li J. Fan Y. Chen Q. (2025). Recent progresses and perspectives of polyethylene biodegradation by bacteria and fungi: a review. *J. Contaminant Hydrol.* 269:104499. 10.1016/j.jconhyd.2025.104499 39787878

[B33] Lin Z. Jin T. Zou T. Xu L. Xi B. Xu D. et al.. (2022). Current progress on plastic/microplastic degradation: fact influences and mechanism. *Environ. Pollut.* 304:119159. 10.1016/j.envpol.2022.119159 35304177

[B34] Lu F. Chao J. Zhao X. Betchem G. Ding Y. Yang X. et al.. (2022). Enhancing protease activity of *Bacillus subtilis* using UV-laser random mutagenesis and high-throughput screening. *Proc. Biochem.* 119 119–127. 10.1016/j.procbio.2022.05.018

[B35] Lyu L. Fang K. Huang X. Tian X. Zhang S. (2024). Polyethylene is degraded by the deep-sea Acinetobacter venetianus bacterium. *Environ. Chem. Lett.* 22 1591–1597. 10.1007/s10311-024-01708-4

[B36] Mao Z. Yu C. Xin L. (2015). Enhancement of phenol biodegradation by Pseudochrobactrum sp. through ultraviolet-induced mutation. *IJMS* 16 7320–7333. 10.3390/ijms16047320 25837630 PMC4425019

[B37] Maroof L. Khan I. Hassan H. Azam S. Khan W. (2022). Microbial degradation of low density polyethylene by Exiguobacterium sp. strain LM-IK2 isolated from plastic dumped soil. *World J. Microbiol. Biotechnol.* 38:197. 10.1007/s11274-022-03389-z 35989357

[B38] Marqués A. M. Burgos-Díaz C. Aranda F. J. Teruel J. A. Manresa À Ortiz A. et al.. (2012). Sphingobacterium detergens sp. nov., a surfactant-producing bacterium isolated from soil. *Int. J. Syst. Evol. Microbiol.* 62 3036–3041. 10.1099/ijs.0.036707-0 22307508

[B39] Montazer Z. Habibi-Najafi M. B. Mohebbi M. Oromiehei A. (2018). Microbial degradation of UV-pretreated low-density polyethylene films by novel polyethylene-degrading bacteria isolated from plastic-dump soil. *J. Polym. Environ.* 26 3613–3625. 10.1007/s10924-018-1245-0

[B40] Najar-Almanzor C. E. González-Díaz R. L. García-Cayuela T. Carrillo-Nieves D. (2025). Adaptation and bioremediation efficiency of UV-mutagenized microalgae in undiluted agro-industrial effluents from mexico. *Environments* 12:307. 10.3390/environments12090307

[B41] Nakai S. Saeki T. (1964). Induction of mutation by photodynamic action in *Escherichia coli*. *Genet. Res.* 5 158–161. 10.1017/S0016672300001105

[B42] Ojha N. Pradhan N. Singh S. Barla A. Shrivastava A. Khatua P. et al.. (2017). Evaluation of HDPE and LDPE degradation by fungus, implemented by statistical optimization. *Sci. Rep.* 7:39515. 10.1038/srep39515 28051105 PMC5209683

[B43] Phillips J. H. Brown D. M. (1967). “The mutagenic action of hydroxylamine,” in *Progress in Nucleic Acid Research and Molecular Biology*, ed. Cohn W. E. (Amsterdam: Elsevier), 349–368. 10.1016/S0079-6603(08)60956-3

[B44] Purohit A. Cochereau B. Sarkar O. Rova U. Christakopoulos P. Antonopoulou I. et al.. (2025). Polyethylene biodegradation: a multifaceted approach. *Biotechnol. Adv.* 82:108577. 10.1016/j.biotechadv.2025.108577 40185175

[B45] Ren H. X. Li G. F. Chen L. Y. Wang M. L. Zhao N. Liu J. M. et al.. (2023). Study on the degradation of low-rank coal by a Sphingobacterium strain. *Coal Conversion* 46 28–35. (In Chinese). 10.19726/j.cnki.ebcc.202302004

[B46] Restrepo-Flórez J.-M. Bassi A. Thompson M. R. (2014). Microbial degradation and deterioration of polyethylene – A review. *Int. Biodeterioration Biodegrad.* 88 83–90. 10.1016/j.ibiod.2013.12.014

[B47] Roager L. Sonnenschein E. C. (2019). Bacterial candidates for colonization and degradation of marine plastic debris. *Environ. Sci. Technol.* 53 11636–11643. 10.1021/acs.est.9b02212 31557003

[B48] Satti S. M. Castro-Aguirre E. Shah A. A. Marsh T. L. Auras R. (2021). Genome annotation of Poly(lactic acid) degrading *Pseudomonas aeruginosa*, Sphingobacterium sp. and Geobacillus sp. *IJMS* 22:7385. 10.3390/ijms22147385 34299026 PMC8305213

[B49] Shapiro T. N. Manucharova N. A. Lobakova E. S. (2022). Activity of alkanmonooxygenase *alk*B gene in strains of hydrocarbon-oxidizing bacteria isolated from petroleum products. *Vestn. VOGiS* 26 575–582. 10.18699/VJGB-22-70 36313823 PMC9556310

[B50] Singh P. Nagarajan A. Chua K. O. Ting A. S. Y. (2023). Biodegradation of low-density polyethylene by novel halophilic bacteria from mangrove ecosystem. *Bioremed. J.* 28 579–590. 10.1080/10889868.2023.2297181

[B51] Song K. Liu J. Zhou W. Zhang F. Fan L. Zhu D. et al.. (2025). Screening, UV mutagenesis, and exploration of enzymatic degradation mechanisms in the highly efficient polyurethane-degrading *Bacillus* sp. J-11. *J. Appl. Microbiol.* 136:lxaf224. 10.1093/jambio/lxaf224 40899743

[B52] Tareen A. Saeed S. Iqbal A. Batool R. Jamil N. (2022). Biodeterioration of microplastics: a promising step towards plastics waste management. *Polymers* 14:2275. 10.3390/polym14112275 35683947 PMC9182643

[B53] Tiwari N. Santhiya D. Sharma J. G. (2024). Significance of landfill microbial communities in biodegradation of polyethylene and nylon 6,6 microplastics. *J. Hazardous Mater.* 462:132786. 10.1016/j.jhazmat.2023.132786 37871442

[B54] Van Beilen J. B. Funhoff E. G. (2007). Alkane hydroxylases involved in microbial alkane degradation. *Appl. Microbiol. Biotechnol.* 74 13–21. 10.1007/s00253-006-0748-0 17216462

[B55] Wanapat M. Muslykhah U. Matra M. Dagaew G. Suriyapha C. Sommai S. et al.. (2025). Biodegradation of low-density polyethylene plastics by cellulolytic *Pseudomonas aeruginosa* isolated from the rumen of Swamp buffalo and the in vitro end-product characteristics. *Environ. Technol. Innovat.* 38:104175. 10.1016/j.eti.2025.104175

[B56] Wang J. Guo X. Xue J. (2021). Biofilm-developed microplastics as vectors of pollutants in aquatic environments. *Environ. Sci. Technol.* 55 12780–12790. 10.1021/acs.est.1c04466 34553907

[B57] Wang L. Luo X. Tang Y. (2025). Highly efficient nitrate and carbon removal from drinking water by aerobic denitrifying mutant strain LWL53-C256: strain isolation, ultraviolet mutagenesis, and mechanism. *J. Environ. Chem. Eng.* 13:118275. 10.1016/j.jece.2025.118275

[B58] Wasmund K. Burns K. A. Kurtböke D. I. Bourne D. G. (2009). Novel alkane hydroxylase gene (*alkB*) diversity in sediments associated with hydrocarbon seeps in the Timor Sea, Australia. *Appl. Environ. Microbiol.* 75 7391–7398. 10.1128/AEM.01370-09 19820158 PMC2786413

[B59] Xia Q. H. You Z. Y. Zhang P. Zhang L. G. Xu X. F. Tang Y. C. (2017). Screening of high-efficiency petroleum hydrocarbon degrading strains by microwave and ultraviolet mutagenesis. *Water Treat. Technol.* 43 36–41. (In Chinese). 10.16796/j.cnki.1000-3770.2017.04.009

[B60] Xiong Z. Chen X. Zou Z. Peng L. Zou L. Liu B. et al.. (2025). Improving efficiency of bacterial degradation of polyethylene microplastics using atmospheric and room temperature plasma mutagenesis. *Bioresour. Technol.* 418:131930. 10.1016/j.biortech.2024.131930 39631542

[B61] Yang G. Quan X. Shou D. Guo X. Ouyang D. Zhuang L. (2025). New insights into microbial degradation of polyethylene microplastic and potential polyethylene-degrading bacteria in sediments of the Pearl River Estuary, South China. *J. Hazardous Mater.* 486:137061. 10.1016/j.jhazmat.2024.137061 39764953

[B62] Yang H. Zhang B. Wu Z. Pan J. Chen L. Xiu X. et al.. (2024). Synergistic application of atmospheric and room temperature plasma mutagenesis and adaptive laboratory evolution improves the tolerance of *Escherichia coli* to L-cysteine. *Biotechnol. J.* 19:2300648. 10.1002/biot.202300648 38403408

[B63] Yu L. Li F. Ni J. Qin X. Lai J. Su X. et al.. (2024). UV-ARTP compound mutagenesis breeding improves macrolactins production of *Bacillus siamensis* and reveals metabolism changes by proteomic. *J. Biotechnol.* 381 36–48. 10.1016/j.jbiotec.2023.12.011 38190850

[B64] Yu Q. Li Y. Wu B. Hu W. He M. Hu G. (2020). Novel mutagenesis and screening technologies for food microorganisms: advances and prospects. *Appl. Microbiol. Biotechnol.* 104 1517–1531. 10.1007/s00253-019-10341-z 31919586

[B65] Zhang P. You Z. Chen T. Zhao L. Zhu J. Shi W. et al.. (2022). Study on the breeding and characterization of high-efficiency oil-degrading bacteria by mutagenesis. *Water* 14:2544. 10.3390/w14162544

[B66] Zhao X. Hussain M. H. Mohsin A. Liu Z. Xu Z. Li Z. et al.. (2024). Mechanistic insight for improving butenyl-spinosyn production through combined ARTP/UV mutagenesis and ribosome engineering in *Saccharopolyspora pogona*. *Front. Bioeng. Biotechnol.* 11:1329859. 10.3389/fbioe.2023.1329859 38292303 PMC10825966

[B67] Zutterling C. Mursalimov A. Talhaoui I. Koshenov Z. Akishev Z. Bissenbaev A. K. et al.. (2018). Aberrant repair initiated by the adenine-DNA glycosylase does not play a role in UV-induced mutagenesis in *Escherichia coli*. *PeerJ* 6:e6029. 10.7717/peerj.6029 30568855 PMC6286661

