# Supplementary material for: Synergistic UV and acridine orange mutagenesis enhances polyethylene biodegradation by Sphingobacterium prati BS2
Source: Front Microbiol. 2026 Mar 12;17:1730975. doi: 10.3389/fmicb.2026.1730975 (PMC13017924; doi:10.3389/fmicb.2026.1730975)
Supplement: Supplementary file 1 [file Data_Sheet_1.docx]

Supplementary Materials and Methods

**S1 Materials**

This study focused on a laboratory-preserved polyethylene (PE)-degrading bacterium, *Sphingobacterium prati* BS2, isolated from cotton field soil with residual plastic mulch. The genus *Sphingobacterium* has been reported for its ability to degrade recalcitrant compounds (Montazer *et al*., 2018; Kim *et al*., 2003; Bandopadhyay *et al*., 2020; Satti *et al*., 2021).

**Major instruments included**: a laminar flow hood (SW-CJ-1B, Suzhou Antai), a high-pressure steam sterilizer (LDZX-50FB, Shanghai Shen'an), an incubator shaker (THZ-100, Shanghai YETUO), an analytical balance (FA2004, Shanghai Puchun), centrifuges and pipettes (Eppendorf), a constant temperature incubator (DNP-9052, Shanghai Jinghong), a UV-Vis spectrophotometer (Model 721, Shanghai Jinghua), a microwave oven (Midea Group), a vortex mixer, a scanning electron microscope (SU8010, Hitachi, Japan), and a Fourier-transform infrared spectrometer (Vertex 70v, Bruker).

**All chemicals were of analytical grade.** PE mulch, PE powder, sodium chloride (NaCl), ammonium sulfate ((NH₄)₂SO₄), iodine (I₂), potassium iodide (KI), tryptone, agar, potassium dihydrogen phosphate (KH₂PO₄), dipotassium hydrogen phosphate (K₂HPO₄), magnesium sulfate heptahydrate (MgSO₄·7H₂O), calcium chloride dihydrate (CaCl₂·2H₂O), and zinc sulfate heptahydrate (ZnSO₄·7H₂O) were purchased from commercial chemical reagent companies.

**LB Medium** (g·L⁻¹): Tryptone 10 g, Yeast Extract 5 g, NaCl 5 g, pH 7.0; for solid medium, 18 g agar was added.

**Mineral Salt Medium** (MSM) (g·L⁻¹): NaCl 0.005 g, FeSO₄·7H₂O 0.002 g, ZnSO₄·7H₂O 0.002 g, CuSO₄ 0.002 g, CaCl₂·2H₂O 0.002 g, MnSO₄·7H₂O 0.001 g, (NH₄)₂SO₄ 1.65 g, MgSO₄·7H₂O 0.7 g, KH₂PO₄ 0.7 g, K₂HPO₄ 0.7 g; for solid medium, 18 g agar and 1 g PE powder were added.

Key solutions used in the experiments included 1% (w/v) acridine orange, 2% (w/v) hydroxylamine hydrochloride, and iodine–potassium iodide developing solution. All solutions were prepared according to standard protocols, then followed by filter-sterilization (or storage protected from light) and kept at 4 °C until use.

**S2 Methods**

**Strain Activation.** The cryopreserved BS2 strain was thawed at 37 ℃, and 0.1 mL of the suspension was spread onto an LB solid medium plate. The plate was incubated inverted at 30 °C for 48 h. Five single colonies were selected, suspended in 5 mL of sterile water, and vortexed thoroughly. Then, 1 mL of the homogeneous suspension was inoculated into 50 mL of LB liquid medium and cultured at 30 ℃ with shaking at 150 rpm for 24 h to obtain the seed culture. The seed culture was transferred into fresh LB medium at a 5% inoculum size and cultivated under the same conditions. The optical density at 600 nm (OD₆₀₀) was measured hourly, using uninoculated medium as the blank control. The experiment was performed in triplicate, and the growth curve was plotted to determine the logarithmic growth phase.

**Determination of Mutagenesis Conditions.** The tolerance of the wild-type strain BS2 to various mutagens was first evaluated through preliminary experiments: gradually increasing gradients of UV irradiation time, microwave power-time combinations, and chemical mutagen concentrations were applied to treat the logarithmic-phase BS2 bacterial suspension. The survival rate of each treatment group was determined to define the range of mutagenic intensities tolerable by the strain.

Mutagenesis gradients were established to ensure optimal conditions with a lethality rate ranging from 80% to 95%. The mutagenesis parameters—including irradiation time, power, and chemical concentrations—were determined based on previous research (He *et al*., 2016) combined with preliminary experiments conducted on wild-type BS2.

**UV mutagenesis:** Following classical microbial mutagenesis protocols, a low-pressure mercury lamp with a wavelength of 254 nm was employed (Wang *et al*., 2025), irradiation distance 30 cm. Preliminary tests showed survival decreased across 10–50 s, adopted as time gradient.

**Microwave mutagenesis:** Pre-experiments at three power levels (300, 500, 800 W) with multiple exposure times to identify suitable power–time combinations (Feng *et al*., 2019).

**Chemical mutagenesis:** Acridine orange (0.030%–0.050%) and hydroxylamine hydrochloride (0.001%–0.009%) were applied based on literature doses (Tian *et al*., 2023; Wang *et al*., 2006). Gradient lethality confirmed by pre-experiments. Lethality rate was calculated as (Yu *et al*., 2024):


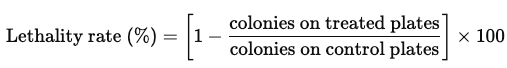


Treatments yielding lethality rates between 80% and 95% were selected for subsequent screening of positive mutants (Wang *et al*., 2024).

**Screening of Positive Mutants.** PE powder was sterilized by UV for 24 h with turning every 3 h, and PE films were sequentially soaked in 2% SDS and 75% ethanol, each for 4 h, followed by an additional 30 min on each side. MSM with pretreated PE as the sole carbon source was prepared. Bacterial suspensions were inoculated onto selective PE plates and non-selective LB plates, incubated at 30 °C for 10 days. Positive mutation rate was calculated as:


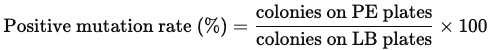


**Screening of Mutant Strains.** Strains grown on the PE selection plates were transferred into LB liquid medium and incubated at 30 °C with shaking at 150 rpm until the logarithmic growth phase was reached. The culture was then harvested, and the bacterial suspension was adjusted to a concentration of 1 × 10⁸ CFU/mL using sterile physiological saline. Wells were created on mineral salt agar plates containing PE powder using a sterile Oxford cup (inner diameter  6 mm, outer diameter  8 mm, height  10 mm), and 100 µL of the bacterial suspension was accurately added into each well. The plates were placed right-side up in an incubator at 30 °C with a relative humidity of 55 ± 5% for 24 h. Thereafter, 4 mL of an iodine-potassium iodide staining solution was added to each plate, followed by incubation at room temperature in the dark for 10 min. The diameter of the clear zone was measured using a vernier caliper along two perpendicular directions; the maximum and minimum values were recorded, and the mean was calculated. The strain exhibiting the largest mean clear-zone diameter was selected. The selected strains were successively subcultured on PE plates for 20 generations, and the degradation-zone diameter was examined every generation to screen for stable strains (Tu *et al*., 2016; Zhang *et al*., 2023; Sun *et al*., 2024; Yu *et al*., 2024). The stable strains were inoculated into MSM medium containing PE film and incubated statically at 30 °C for 50 days. During this period, OD₆₀₀ and pH were measured every 7 days, and multi-scale analyses were performed after the incubation. After 50 days, the PE film pieces were stained with 0.1% crystal violet for 20 min, washed with PBS and 2% SDS, and then dissolved in 96% ethanol for 6 h, after which the OD₅₇₀ was determined. Sterile PE films were used as controls.

**PE Characterization.** PE fragments (MPs) recovered from the culture medium and PE films subjected to biofilm detection were washed three times with 75% ethanol and sterile water, then dried at 50 ℃. The weight loss rate was calculated as follows:


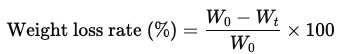


The surface morphology of the samples was observed using field emission scanning electron microscopy (FE-SEM), and changes in chemical composition were analyzed by attenuated total reflectance Fourier-transform infrared spectroscopy (ATR-FTIR). Based on the ATR-FTIR results, the hydroxyl index (HI) and carbonyl index (CI) were calculated using the following equations:

HI = A₃₇₅₀–₃₄₀₀ / A₁₄₉₅–₁₄₁₅

CI = A₁₈₅₀–₁₆₅₀ / A₁₄₉₅–₁₄₁₅

(Aₓ–y indicates integrated absorbance over the specified wavenumber range.)

Statistical analysis was performed using one-way analysis of variance (ANOVA) and the least significant difference (LSD) test in the R programming environment.

Supplementary Figure


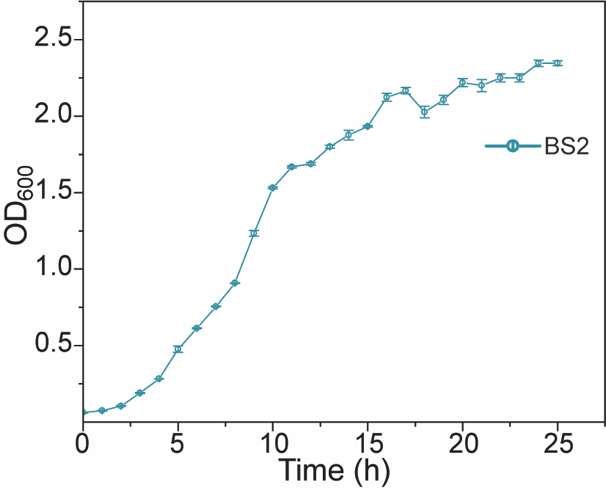


Fig. S1 Growth curve of the wild-type *Sphingobacterium prati* BS2.

Data are presented as mean ± SD (n = 3).

**Reference**

Bandopadhyay, S., Liquet Y González, J. E., Henderson, K. B., Anunciado, M. B., Hayes, D. G., and DeBruyn, J. M. (2020). Soil Microbial Communities Associated With Biodegradable Plastic Mulch Films. Front. Microbiol. 11, 587074. doi: 10.3389/fmicb.2020.587074

Feng, W., Liang, J., Wang, B., and Chen, J. (2019). Improvement of kojic acid production in Aspergillus oryzae AR-47 mutant strain by combined mutagenesis. Bioprocess Biosyst Eng 42, 753–761. doi: 10.1007/s00449-019-02079-9

Kim, B. C., Sohn, C. K., Lim, S. K., Lee, J. W., and Park, W. (2003). Degradation of polyvinyl alcohol by Sphingomonas sp. SA3 and its symbiote. J IND MICROBIOL BIOTECHNOL 30, 70–74. doi: 10.1007/s10295-002-0010-4

Montazer, Z., Habibi-Najafi, M. B., Mohebbi, M., and Oromiehei, A. (2018). Microbial Degradation of UV-Pretreated Low-Density Polyethylene Films by Novel Polyethylene-Degrading Bacteria Isolated from Plastic-Dump Soil. J Polym Environ 26, 3613–3625. doi: 10.1007/s10924-018-1245-0

Satti, S. M., Castro-Aguirre, E., Shah, A. A., Marsh, T. L., and Auras, R. (2021). Genome Annotation of Poly(lactic acid) Degrading Pseudomonas aeruginosa, Sphingobacterium sp. and Geobacillus sp. IJMS 22, 7385. doi: 10.3390/ijms22147385

Sun, J., Zhang, Z., Liu, J., and Zhang, S. (2024). UV-ARTP combined mutagenesis selection of lignin-degrading strain and enzymatic characterization of the resultant organisms. Food Bioscience 60, 104478. doi: 10.1016/j.fbio.2024.104478

Tian, H., Cai, Z., Zhang, Y., and Zheng, Q. (2023). Chemical mutation of Bacillus mucilaginosus genes to enhance the bioleaching of vanadium-bearing shale. Biochemical Engineering Journal 197, 108962. doi: 10.1016/j.bej.2023.108962

Tu, R., Jin, W., Wang, M., Han, S., Abomohra, A. E.-F., and Wu, W.-M. (2016). Improving of lipid productivity of the biodiesel promising green microalga Chlorella pyrenoidosa via low-energy ion implantation. J Appl Phycol 28, 2159–2166. doi: 10.1007/s10811-015-0783-2

Wang, Z., Chen, Y., Hong, Y., Li, L., Zhang, Y., Wang, C., et al. (2025). From laboratory-level construction to pilot-level validation: Utilization of compound mutagenic bacterial agent for efficient treatment of kitchen waste. Journal of Environmental Chemical Engineering 13, 116441. doi: 10.1016/j.jece.2025.116441

Yu, L., Li, F., Ni, J., Qin, X., Lai, J., Su, X., et al. (2024). UV-ARTP compound mutagenesis breeding improves macrolactins production of Bacillus siamensis and reveals metabolism changes by proteomic. Journal of Biotechnology 381, 36–48. doi: 10.1016/j.jbiotec.2023.12.011

Zhang, A., Ma, Y., Deng, Y., Zhou, Z., Cao, Y., Yang, B., et al. (2023). Enhancing Protease and Amylase Activities in Bacillus licheniformis XS-4 for Traditional Soy Sauce Fermentation Using ARTP Mutagenesis. Foods 12, 2381. doi: 10.3390/foods12122381

He, H., Liu, P., Luo, B. X., Zhang, E. L., Tao, Z. Y., Qi, S. M., et al. (2016). Screening and mutagenic breeding of an actinomycete strain degrading starch-filled polyethylene. Journal of Sichuan Normal University (Natural Science Edition) 39, 408–413. (In Chinese)

Wang, G. Q., Ren, Y. Z., Zhang, J., Ma, X., Zheng, R., Su, J. Y., et al. (2024). Screening of aroma-enhancing and high-yield alcohol-producing selenium-enriched yeast by ARTP mutagenesis. Science and Technology of Food Industry 45, 146–156. doi: 10.13386/j.issn1002-0306.2023110161. (In Chinese)

Wang, S. N., Gao, H., Sun, J. L., and Song, C. W. (2006). Study on the mutagenic effects of different mutagens on Acetobacter. Journal of Henan Institute of Science and Technology (Natural Science Edition), 5–8. (In Chinese)
